# The Therapist from Within: A Psychodynamic–Phenomenological Comparison of the Therapist’s Lived Experience in Two Anxiety-Laden Psychotherapeutic Encounters

**DOI:** 10.3390/bs16101752

**Published:** 2026-09-25

**Authors:** Lida-Alkisti Xenaki

**Affiliations:** First Department of Psychiatry, Eginition Hospital, Medical School, National and Kapodistrian University of Athens, 11528 Athens, Greece; lxenaki@med.uoa.gr

**Keywords:** psychodynamic psychotherapy, phenomenology, therapist lived experience, intersubjectivity, object relations, shame, guilt

## Abstract

The therapist’s lived experience remains comparatively underrepresented in psychotherapy research despite its importance for clinical understanding and therapeutic decision-making. Drawing on a psychodynamic–phenomenological perspective, this qualitative study examines how the therapist’s experience can function as a source of clinical knowledge through the comparative analysis of two anonymized psychotherapeutic cases. The psychotherapies were conducted within a psychodynamic framework, while their subsequent analysis employed phenomenological reflection on the therapist’s embodied, affective, and relational participation in the therapeutic encounter. Although both patients presented with prominent anxiety accompanied by control, perfectionism, and inhibition, they generated markedly different therapeutic fields. In the first case, organized predominantly around narcissistic vulnerability and shame, the therapist experienced fragile proximity, subtle discouragement, and recurrent loss of emotional contact. In the second, organized around guilt, harsh superego functioning, and fear of criticism, the therapist’s experience was characterized by vigilance, careful pacing, and the need to contain affect without intensifying self-attack. These contrasting experiential configurations revealed distinct transferential–countertransferential organizations that were not immediately evident from symptom presentation alone. The findings support the integration of psychodynamic concepts with phenomenological reflection and suggest that the therapist’s first-person perspective constitutes a meaningful source of qualitative clinical knowledge, enriching contemporary first-person approaches to psychotherapy research.

## 1. Introduction

Psychotherapy is an intersubjective encounter involving the subjective worlds of both patient and therapist. The patient attempts to articulate thoughts, emotions, fantasies, and conflicts, while the therapist listens, receives, contains, and interprets what emerges (Foehl, 2020; Opland & Torrico, 2024; Stolorow & Atwood, 1984). These subjective worlds continuously shape one another within a shared relational field in which meanings are experienced and transformed (Lundh & Falkenström, 2019; Zahavi & Loidolt, 2022).

Psychotherapy research has traditionally privileged the patient’s symptoms, developmental history, psychopathology, and treatment outcomes, while the therapist’s lived experience has remained comparatively underrepresented as a legitimate source of clinical knowledge (Fuchs et al., 2019; Lundh & Falkenström, 2019). Still, every therapeutic encounter simultaneously unfolds within the therapist’s own experiential world, shaping perception, bodily awareness, emotional resonance, reflection, imagination, and clinical judgment (Neubauer et al., 2019). Recent developments in phenomenological psychopathology, psychoanalytic phenomenology, and first-person clinician research have increasingly challenged this asymmetry. These approaches revisit classical phenomenological psychiatry, particularly Jaspers’ emphasis on the disciplined understanding of subjective experience (Jaspers, 1968, 1997), while recognizing therapist subjectivity as an integral dimension of the therapeutic process (Atwood & Stolorow, 2014; Stanghellini et al., 2019; Xenaki, 2026).

Within psychodynamic psychotherapy, therapist subjectivity has long been conceptualized through transference and countertransference, with contemporary approaches viewing the therapist’s affective participation as potentially informative about the developing relational field (Freud, 1912/1958; Greenson, 1967; Orange et al., 1997). Countertransference may therefore provide an important avenue through which aspects of the patient’s internal world become experientially accessible. Building upon this tradition, Stolorow and Atwood (1984) proposed an intersubjective psychoanalytic perspective in which clinical understanding arises within the reciprocal interaction between therapist and patient. Similarly, Chessick (1992) emphasized that analytic understanding necessarily develops through the therapist’s reflective awareness of his or her own lived participation in the analytic process. Contemporary psychoanalytic phenomenology has further extended this position by viewing therapeutic knowledge as emerging through the unfolding relational field itself, where subjective experience becomes both the medium and the object of clinical understanding (Atwood & Stolorow, 2014; Foehl, 2020; Stanghellini et al., 2019).

Moreover, psychoanalytic theory has demonstrated that patients with apparently similar symptom presentations may be organized by fundamentally different unconscious relational configurations. From Freud’s early formulations of narcissism, anxiety, and transference to later developments in object relations and self-psychology, authors have consistently shown that similar symptoms may conceal markedly different modes of relating, defensive organizations, and developmental conflicts (Freud, 1914/1957, 1926/1959, 1924/1961, 1923/1961; Strachey, 1934; Loewald, 1960; Kernberg, 1975; Kohut, 1977; Rosenfeld, 1987). Particularly important in this regard is the distinction between shame and guilt, where shame primarily concerns exposure and threats to self-cohesion, while guilt concerns responsibility, conscience, and reparation (Morrison, 1984; Gillman, 1990; Lansky, 1994).

Phenomenological psychopathology complements this psychodynamic perspective by focusing on how these relational processes are actually lived. Phenomenology seeks to describe the experiential structure of psychotherapy in terms of atmosphere, embodiment, temporality, affective resonance, interpersonal distance, tension, hesitation, burden, and relief as they emerge within the therapeutic encounter (Fuchs et al., 2019; Jaspers, 1968, 1997; Zahavi & Loidolt, 2022). As Foehl (2020) argues, remaining close to lived experience allows the depth of the psychotherapeutic process to become visible without prematurely reducing it to conceptual explanation. Recent methodological developments have increasingly advocated the integration of phenomenological description and psychodynamic interpretation. In particular, Stanghellini’s (2019) PHD method integrates phenomenology, hermeneutics, and psychodynamics, moving from disciplined description of lived experience toward exploration of meaning and psychodynamic contextualization. Likewise, phenomenologically informed psychotherapy research suggests that disciplined examination of first-person clinical experience can illuminate dimensions of the therapeutic process that remain difficult to access through symptom-focused or outcome-oriented methodologies (Daly & Gallagher, 2019; Daly et al., 2024).

The present study is situated at the intersection of psychodynamic psychotherapy, phenomenological inquiry, and first-person clinician research. Through a qualitative comparison of two anonymized psychodynamic psychotherapies, it explores how the therapist’s lived experience becomes a source of clinical understanding when examined phenomenologically and interpreted psychodynamically.

## 2. Materials and Methods

This study employed a qualitative, reflexive methodology grounded in clinician first-person inquiry (Neubauer et al., 2019; Stanghellini, 2019). The clinical material comprised two psychotherapeutic cases purposively selected for their comparative and illustrative value. Both patients initially presented with prominent anxiety, high levels of functioning, perfectionistic tendencies, and reliance on control. However, over time they generated markedly different therapeutic atmospheres and countertransferential experiences. Their comparative value lay in the gradual emergence of distinct affective and relational organizations beneath superficially similar clinical presentations. Personality formulations were therefore used as psychodynamic organizing concepts rather than exhaustive categorical diagnoses. Consistent with phenomenologically oriented clinical research, the two-case design was intended to illuminate distinctive experiential configurations and support phenomenological and clinical differentiation (Sandsten et al., 2022).

Both cases involved ongoing, once-weekly psychodynamic psychotherapy, and the current presentation refers to the first year of treatment. Written informed consent for publication of anonymized clinical material was obtained from both individuals described. Identifying particulars have been changed or disguised where necessary to preserve anonymity while retaining the psychodynamic organization and phenomenological significance of the clinical material.

The therapist occupied the dual role of clinician and investigator (Salifu, 2025). Accordingly, the clinical material consists of retrospective accounts informed by therapeutic participation and subsequent theoretical reflection rather than verbatim reproductions of psychotherapy sessions. The reconstruction of the clinical material was informed by contemporaneous session notes, which served as clinical records supporting retrospective reflection. No audio or video recordings of the sessions were available. Sessions were conducted within a psychodynamic framework, and their subsequent examination integrated phenomenological reflection, hermeneutic interpretation, and psychodynamic formulation (Aleksandrov, 1997; Stanghellini, 2019). Specifically, the analysis was informed by the integrative logic of Stanghellini’s (2019) PHD method, adapting its dialogue among phenomenology, hermeneutics, and psychodynamics to therapist-focused first-person inquiry. The cases are presented as reconstructed clinical narratives, consistent with contemporary psychoanalytic case writing and phenomenological qualitative research (Aron, 2000; Peterlini & Rathgeb, 2026; Sieck et al., 2025).

Within this reflexive framework, therapist experiences were regarded as clinically meaningful aspects of the therapeutic encounter. Their interpretation remained provisional and open to alternative formulations, and therapist subjectivity was approached as an object of disciplined reflection throughout the analysis of the clinical material (Neubauer et al., 2019; Stanghellini et al., 2019; Stolorow & Atwood, 1984).

### 2.1. Psychodynamic Therapeutic Framework

Both psychotherapies were conducted by the author, a psychiatrist–psychotherapist and member of the International Psychoanalytical Association. Treatment followed established psychodynamic principles, including free association, evenly suspended attention, and the exploration of unconscious meanings as they emerged within the transference-countertransference relationship (Abrahams & Rohleder, 2021; Greenson, 1967). Therapeutic work focused on the interpretation of unconscious conflict, defensive processes, resistance, and transference (Greenson, 1967). Interpretations arose from the unfolding psychotherapeutic dialogue and were timed according to each patient’s capacity to tolerate affect, sustain reflective functioning, and integrate emerging meanings (Chessick, 1992; Orange et al., 1997; Stolorow & Atwood, 1984). Both treatments were conducted under regular supervision by a Training and Supervising Analyst of the International Psychoanalytical Association with longstanding scholarly and clinical engagement in phenomenological psychiatry.

### 2.2. Examination of the Clinical Material

The clinical material was examined in three complementary phases. First, each case was revisited phenomenologically from the therapist’s first-person perspective, attending to atmosphere, embodiment, temporality, affective resonance, interpersonal distance, and shifts in emotional participation before explanatory concepts were introduced. Second, a reflexive–hermeneutic examination considered how these experiences acquired meaning over time, taking into account retrospective understanding, alternative interpretations, and theoretical commitments. Third, these experiential observations were interpreted psychodynamically through concepts including transference, countertransference, object relations, shame, guilt, defensive organization, developmental conflict, affective symbolization, and superego functioning^1^ (Greenson, 1967; Orange et al., 1997).

The clinician–investigator dual role required particular reflexive attention. Regular supervision provided an additional perspective from which the therapist’s immediate impressions, countertransferential responses, and emerging formulations could be reconsidered. In this sense, supervision introduced a third reflective position into the examination of the therapeutic dyad, helping to distinguish participation in the relational field from subsequent interpretation of it. Furthermore, supervision provided a structured context in which experiential hypotheses could be questioned, revised, and considered alongside alternative formulations.

## 3. Results

### 3.1. Case One: Narcissistic Personality Traits and Prominent Anxiety

From the beginning of treatment, I was struck by a recurring paradox. The patient entered sessions composed, articulate, and psychologically minded. However, genuine emotional contact often seemed to recede just as it began to deepen. I was frequently left with the impression that something important had almost taken shape between us before quietly dissolving. The therapeutic process was therefore characterized by subtle withdrawal, leaving a persistent sense of incompleteness and unfinished contact.

The patient was a highly functioning adult woman in her 30s with longstanding anxiety and prominent narcissistic personality traits. She maintained considerable professional and family responsibilities and strongly identified with competence, self-sufficiency, and the ability to support others. She sought treatment with the wish to develop greater self-understanding and a more secure sense of self, while seeking greater capacity to manage longstanding anxiety and intermittent episodes of stress that left her feeling overwhelmed. Over time, marked sensitivities around dependency, inadequacy, bodily exposure, femininity, and sexuality became increasingly apparent beneath this capable presentation. Her self-esteem appeared closely tied to performance and composure, while anxiety and control increasingly came to be understood as protecting against shame, helplessness, and the experience of needing another person.

Her developmental background was organized around a strong paternal ideal of achievement, authority, and duty. The father occupied a prominent psychological position within the family and was experienced as demanding and closely associated with standards of effectiveness. The mother was experienced as capable and devoted but more strongly identified with support, accommodation, and responsibility. Within this family structure, competence appeared to confer value, whereas dependency and vulnerability were less easily integrated into a positive sense of self.

Sibling relationships were marked by comparison and competition. The patient described longstanding sensitivity to recognition, ranking, and the possibility of being displaced by others. These experiences appeared to contribute to a recurring need to establish worth while simultaneously protecting herself from the shame of not being preferred, admired, or regarded as sufficiently capable. Similar patterns later became visible in intimate relationships through oscillations between admiration, disappointment, comparison, and devaluation.

A further developmental theme involved femininity and sexuality. Earlier experiences of moralized or shaming responses to sexual development seemed to contribute to a conflict between desire and exposure. In adulthood, longing for tenderness and recognition coexisted with withdrawal, abstinence, and the need to preserve control. Bodily and erotic closeness appeared to carry risks of dependency, exposure, judgment, and potential humiliation.

Parenthood introduced another important dimension. A serious medical crisis involving one of her children appeared to reinforce the sense that safety could not be taken for granted and that vigilance was necessary to prevent catastrophe. Parenthood mobilized genuine tenderness and devotion while at the same time intensifying responsibility, control, and difficulty entrusting care to others.

As treatment progressed, I became increasingly aware of occupying an uncertain position within her internal world. At times I experienced myself as helpful, containing, and emotionally available, while at other times this sense of connection quietly receded. Emotionally significant material could become factual; practical concerns displaced affectively charged themes, and appointments were occasionally altered or missed. I experienced an elusive uncertainty, as though my value within the relationship required continual confirmation without ever becoming securely established.

The sessions were often lived as restrained and delicately balanced. Emotional proximity seemed simultaneously invited and inhibited, and I repeatedly noticed a bodily sense of caution. Genuine contact remained possible, but often only briefly. What lingered was not rupture itself but the gradual fading of relational immediacy.

The transference increasingly appeared organized around the wish to be recognized and the fear of becoming dependent upon that recognition. I seemed to become a potentially valuable object whose availability was desired but whose importance had to remain limited enough to protect the patient from the shame of visible dependency. Oscillations between warmth and withdrawal, or between idealization and subtle devaluation, became understandable as ways of regulating relational proximity.

My countertransferential experience was marked by fatigue, discouragement, and subtle self-questioning. I often felt an implicit pressure to remain useful and to demonstrate therapeutic value. Moments in which something meaningful seemed to have been recognized were frequently followed by renewed distance, producing a recurring sense that the treatment was “slipping away” just as emotional contact deepened. At times I felt drawn toward becoming more active, explanatory, or reassuring, which I came to understand as part of the pressure generated within the therapeutic field.

Across different areas of the patient’s life, a recurring polarity emerged between control and tenderness. Achievement, vigilance, and self-sufficiency protected self-esteem, whereas receptivity, dependency, and emotional surrender carried the risk of humiliation. Shame appeared to be the dominant organizing affect, particularly around inadequacy, sexuality, dependency, and the possibility of being seen as less competent than demanded by the internal ideal. Guilt was also present, but more often as duty and harsh self-demand than as the primary affective organizer.

Despite these defensive patterns, the patient possessed substantial psychological resources. She was intelligent, reflective, professionally capable, deeply responsible, and able to sustain long-term commitments. Parenthood revealed a genuine capacity for tenderness and care, and she demonstrated considerable curiosity about psychological meaning. These capacities provided important therapeutic leverage, as qualities previously mobilized in the service of control could gradually support reflection, uncertainty, and greater tolerance of dependency.

Looking back upon the treatment, anxiety appeared increasingly embedded within a broader organization involving narcissistic vulnerability, shame, dependency, and the need to preserve self-cohesion. Closeness could emerge, but only briefly, before being regulated through withdrawal, factualization, or devaluation. The therapeutic challenge was therefore not to dismantle control directly but to create conditions in which need, tenderness, desire, and uncertainty could become more tolerable without immediately being experienced as evidence of weakness or humiliation.

### 3.2. Case Two: Obsessive–Compulsive Personality Traits and Prominent Anxiety

From the outset, the therapeutic atmosphere was marked by emotional intensity and fragility. I frequently experienced a strong sense of responsibility for regulating the pace of the work. I often felt that we were moving through a narrow emotional corridor in which proceeding too quickly risked overwhelming the patient, whereas moving too cautiously risked leaving her alone with an increasingly punitive internal world. This pervasive atmosphere of carefulness became one of the earliest indications of the distinctive organization of the treatment.

The patient was a highly functioning adult woman in her 30s with substantial family, occupational, and social responsibilities. She presented as caring, empathic, conscientious, and deeply invested in the well-being of others. She sought treatment because of progressively worsening anxiety characterized by anticipatory worry, intermittent panic-like episodes, somatic symptoms, fears of losing control, and increasing emotional exhaustion. Gradually, these symptoms appeared to be part of a broader constellation of obsessive–compulsive personality traits.

Her developmental background suggested a family environment in which love, responsibility, and achievement coexisted with considerable emotional restraint. Both parents were experienced as devoted and highly functional, but expressions of anger, disappointment, vulnerability, and conflict were limited. Family relationships emphasized loyalty, self-control, and maintaining harmony, while negative affect often remained unspoken or insufficiently recognized. Other family members had also experienced depressive difficulties, reinforcing the presence of psychological vulnerability within an otherwise high-functioning family system.

As an adult, the patient entered a demanding family-based professional environment in which responsibility was high, but disagreement was difficult to express. Decisions tended to remain concentrated in the older generation, and the patient often felt that her views could be heard without necessarily influencing outcomes. This pattern echoed a broader interpersonal style in which she assumed responsibility readily but struggled to assert dissatisfaction, anger, or personal limits.

Her psychological functioning was increasingly organized around perfectionistic standards, inhibition of anger, and a harsh moralizing superego. Emotional distress was rarely expressed through overt conflict. Instead, it was displaced into bodily tension, autonomic symptoms, hypervigilance, freezing, and severe self-criticism. She tended to minimize the shortcomings of others while directing judgment toward herself. Rest, dependency, self-care, and the expression of frustration could rapidly acquire meanings of selfishness, irresponsibility, or moral failure.

This became particularly visible during periods of increased relational and practical stress. Situations involving responsibility, interpersonal uncertainty, or perceived failure could quickly be experienced as evidence of personal inadequacy, accompanied by intense tearfulness. What struck me most was her difficulty transforming intense affect into something that could be reflected upon before it became overwhelming. Affect seemed to accumulate until it exceeded her capacity for psychological elaboration and emerged as crying, bodily tension, somatic sensations, or emotional freezing. Reflective capacity appeared particularly fragile as affective intensity increased, indicating the need to preserve conditions under which emotional experience could become thinkable rather than simply discharged.

The transference was characterized by a persistent concern that she might prove inadequate in my eyes. My presence was experienced as potentially judging. The wish for support itself often seemed morally problematic, as though dependency confirmed weakness. I gradually came to occupy the position of an observing or potentially disappointed figure before whom emotional disorganization felt deeply exposing. This dynamic became especially visible in moments of silence. When I gently drew attention to the severity of her self-judgment, she would sometimes respond, “I don’t know,” and stop speaking. The silence appeared to protect her from anticipated judgment, shame, or the fear of saying something morally unacceptable.

Her marital relationship provided another context in which these dynamics became apparent. She described a relationship that had begun with affection and mutual investment but had become increasingly disappointing over time. She often experienced her partner as preoccupied with his own needs and less able to recognize her subjective experience. What seemed particularly painful was her own uncertainty about whether she had the right to feel disappointed. Dissatisfaction was quickly followed by guilt and self-doubt.

Parenthood also intensified her sense of responsibility. She became highly preoccupied with maintaining emotional balance among her children and with the possible psychological consequences of differential parental attention, developmental transitions, and differing temperaments. What stood out was the extent to which she experienced herself as responsible for anticipating and preventing imbalance within the family while leaving little room for her own emotional needs.

Her narratives repeatedly reflected this pattern. When describing situations in which she felt pressured, she would often defend or justify the other person almost immediately, then turn criticism toward herself. In psychodynamic terms, these movements appeared to protect relationships by redirecting aggression inward. The same pattern emerged around self-care. After describing a brief period in which she chose rest over an immediate family obligation, she rapidly concluded that she had been selfish and at fault. What was striking was the speed with which an ordinary personal need became transformed into moral accusation. The sequence repeatedly seemed to move from need to self-judgment to closure of reflection.

My countertransferential experience was characterized by vigilance, careful pacing, and a growing sense of protective responsibility. I became aware of monitoring the emotional impact of interpretation, concerned that even useful formulations might intensify her self-criticism. At times, I also felt pressure to provide rapid reassurance or relief. These responses became clinically meaningful insofar as they mirrored the patient’s own urgent wish to escape distress while preserving an internal system organized around control and moral demand.

Looking back upon the treatment, the patient’s personality organization appeared structured around excessive responsibility, harsh superego functioning, chronic guilt, and limited affective symbolization. Anxiety seemed less like an isolated symptom than the affective expression of a conflict between ordinary human needs and an internal authority demanding constant competence, self-control, and self-sacrifice. Obsessive–compulsive defenses, including intellectualization, overcontrol, suppression of anger, moralization, and turning aggression against the self, helped preserve psychological equilibrium while restricting emotional flexibility and self-compassion. The central therapeutic task therefore consisted in preserving the patient’s capacity to think in the face of internal self-attack. The therapeutic relationship first needed to provide sufficient containment for emotional experience to become bearable before it could become meaningful.

### 3.3. Comparative Analysis

Although both patients presented with prominent anxiety and maintained high levels of occupational and interpersonal functioning, the therapeutic relationships evolved into distinctly different experiential fields. These differences became evident not primarily through symptom content but through the way each patient organized the therapeutic encounter and the corresponding countertransferential experience.

The defining atmosphere of the first treatment was one of fragile proximity. Emotional closeness repeatedly emerged only to become attenuated through subtle withdrawal, factualization, or devaluation. The central relational tension concerned recognition and dependency. The patient appeared to long for emotional contact while simultaneously protecting herself from the shame associated with needing it. Consequently, the therapeutic work required maintaining relational continuity without intensifying narcissistic vulnerability or defensive retreat.

By contrast, the second treatment was characterized by fragile containment. Here, emotional contact itself remained available, but affect rapidly exceeded the patient’s capacity for reflection. Rather than distancing from the relationship, she became increasingly vulnerable to emotional flooding, self-condemnation, and reduced capacity for symbolization. The therapeutic challenge therefore consisted less in preserving closeness than in preserving the patient’s capacity to remain psychologically present while difficult affect was experienced.

These contrasting atmospheres reflected different dominant affective organizations. In the first case, anxiety appeared embedded within a narcissistic configuration organized predominantly around shame, self-esteem regulation, and the protection of self-cohesion. In the second, anxiety was more closely organized around guilt, harsh superego functioning, and the transformation of ordinary needs into moral transgression. Although both patients relied on control as a principal defense, its function differed. In the first case, control regulated interpersonal closeness and protected against humiliation, while in the second, it maintained moral order and defended against anticipated self-criticism.

The developmental narratives were consistent with these distinct organizations. The first patient’s history suggested an internal world shaped by ideals of achievement, recognition, and competitive comparison, whereas the second patient’s background reflected the internalization of responsibility, emotional restraint, and conflict avoidance. These developmental themes are understood as contexts within which each patient’s characteristic modes of experiencing self and others gradually became organized.

The countertransference provided one of the clearest points of differentiation. In the first treatment, I was repeatedly drawn into experiences of uncertainty, discouragement, and unstable recognition, mirroring a relational field organized around narcissistic vulnerability. In the second, I experienced vigilance, protective responsibility, and careful pacing, reflecting a field dominated by affective overload and harsh internal evaluation. Considered phenomenologically, these differing experiences functioned as clinically meaningful pathways into each patient’s distinctive organization of experience. Table 1 summarizes the principal phenomenological and psychodynamic differences between the two therapeutic processes, highlighting how comparable anxiety presentations generated distinct relational configurations, technical demands, and therapist experiences.

Together, the two treatments illustrate that anxiety cannot be adequately understood apart from the personality organization within which it is embedded. Similar symptom presentations may conceal fundamentally different experiential worlds, requiring correspondingly different therapeutic attitudes, interpretive timing, and modes of relational participation. Integrating psychodynamic understanding with careful attention to the therapist’s lived experience allows the underlying organization of the therapeutic field to become more fully intelligible.

## 4. Discussion

The present findings support therapist subjectivity as a clinically meaningful source of knowledge when approached reflexively. Across both treatments, the therapist’s experience of uncertainty, vigilance, discouragement, responsibility, or restraint contributed to understanding the organization of the therapeutic field. This is consistent with psychoanalytic and intersubjective approaches to countertransference and relational meaning (Greenson, 1967; Orange et al., 1997; Rosenfeld, 1987; Stolorow & Atwood, 1984).

The comparison further illustrates the complementary value of phenomenological and psychodynamic perspectives. Differences between the two encounters first emerged through atmosphere, bodily participation, emotional pressure, and relational distance and were subsequently understood psychodynamically in terms of conflict, defense, object relations, and affective organization. Phenomenology thus preserved lived experience before explanation, while psychodynamic formulation explored its developmental and relational meanings (Aleksandrov, 1997; Fuchs et al., 2019; Stanghellini et al., 2019). From this perspective, individual symptoms acquire their clinical significance within broader experiential and relational configurations. This resonates with Foehl’s (2020) phenomenological account of psychoanalytic process as a mode of lived participation through which clinical understanding gradually acquires depth rather than appearing fully formed from theoretical interpretation alone.

Noy’s (1982) revision of psychoanalytic affect theory provides an important conceptual bridge between these perspectives. Affect is understood as an organizing process involving bodily, perceptual, cognitive, motivational, communicative, and behavioral dimensions. From this perspective, changes in the therapist’s bodily tension, attentional focus, impulses toward intervention or restraint, and fluctuating capacity to think may themselves express aspects of the patient’s affective organization before these become symbolized or verbally articulated. The findings are likewise compatible with Bion’s (1962, 1970) conception of containment, according to which emotional experience first requires transformation within a receptive relationship before it becomes available for thinking. The therapist’s lived experience may therefore provide access to affective organization at precisely those moments when the patient cannot yet symbolize or reflect upon it.

A further clinically relevant distinction concerns the predominant organization of shame and guilt. Consistent with previous psychoanalytic literature, shame in the first case appeared primarily related to exposure, fear of dependency, and narcissistic vulnerability, whereas guilt in the second case was organized around moral accountability, self-criticism, and the transformation of ordinary needs into perceived personal failure (Anastasopoulos, 1997; Benau, 2017; Morrison, 1984; Lansky, 1994, 1999, 2004). At the same time, the present findings support Pulver’s (1999) caution against treating either affect as an exhaustive explanatory principle. Shame and guilt are better understood as predominant organizing tendencies within broader affective systems that also include anxiety, aggression, defensive operations, and interpersonal regulation. The clinical question, therefore, is how each affect structures the patient’s relationship with self, others, and the therapist.

These findings also carry technical implications. Psychodynamic technique requires sensitivity to the patient’s capacity to tolerate and symbolize affect. Interpretation cannot be separated from the patient’s current capacity for affective integration, a principle emphasized in contemporary psychodynamic psychotherapy (Abrahams & Rohleder, 2021). Likewise, the therapist’s task often involves providing a sufficiently reliable relational environment in which difficult emotional experience can gradually become thinkable rather than immediately defended against or discharged (Bion, 1962, 1970; Winnicott, 1960). Countertransference may inform exploratory clinical hypotheses that acquire meaning through ongoing reflection and therapeutic dialogue (Mendes, 2022). Therapeutic judgment thus emerges as a reflexive process integrating theoretical formulation, clinical experience, emotional awareness, and the patient’s moment-to-moment capacities (Carlson, 2009).

Beyond their comparative value, these two cases provide an experiential basis for considering how an integrative psychodynamic–phenomenological approach may enrich psychotherapeutic work. The argument developed here arises from direct participation in the two therapeutic processes and from reflection on how understanding evolved within the sessions themselves. Quiet experiential phenomena, including hesitation before speaking, subtle bodily caution, unexpected fatigue, narrowing of spontaneity, or uncertainty regarding the timing of interpretation within the sessions, contributed to clinical understanding without being treated as self-evident evidence about the patient. The two cases thus illustrate, from within psychotherapeutic practice, how a psychodynamic–phenomenological perspective may extend beyond formulation to enhance relational attunement, therapeutic responsiveness, and the ongoing refinement of clinical judgment.

Within this process, supervision provides a third reflective perspective from which experiential hypotheses can be examined beyond the immediacy of the transference–countertransference field and differentiated from the therapist’s own assumptions or theoretical expectations. This function is particularly important during training but remains relevant throughout professional practice.

Overall, the specific contribution of this study lies in bringing countertransference, phenomenological reflection, and psychodynamic formulation into structured dialogue within the therapist’s first-person inquiry. Comparing two superficially similar anxiety presentations illustrates how differences in the therapist’s embodied, affective, and relational experience may differentiate therapeutic fields and orient distinct technical responses.

These interpretations are inevitably shaped by the author’s theoretical and clinical formation, combining formal psychoanalytic training with sustained scholarly engagement in phenomenological psychopathology, including doctoral study. This dual orientation informed an experience-near approach attentive to intersubjectivity and the dialogue between natural–scientific and human–scientific modes of understanding. The resulting formulations should therefore be understood as reflexive and theoretically situated rather than exhaustive.

Several limitations should be acknowledged. The study is based on two purposively selected, retrospectively reconstructed case narratives and therefore does not support empirical, diagnostic, or population-level generalization. Although contemporaneous session notes informed the reconstruction, no verbatim recordings were available; selection, memory, theoretical orientation, and emotional salience inevitably influenced the material and its interpretation. Both psychotherapies were ongoing at the time of writing, so the formulations necessarily represent provisional understandings of evolving therapeutic processes. Moreover, the patients’ own first-person perspectives remain mediated through the therapist’s narrative. As the International Psychoanalytical Association (2018) Confidentiality Committee observes, every presentation of clinical material is necessarily shaped by the analyst’s perspective and unconscious participation, making case writing both scientifically valuable and epistemologically provisional. This position is further reflected in contemporary discussions of psychotherapeutic case writing and clinician-research, which emphasize that clinical narratives should be understood as interpretive reconstructions and that psychoanalytic discussions of identical clinical material frequently produce differing yet equally plausible interpretations (Aron, 2000; Pulver, 1999; Salifu, 2025; Sieck et al., 2025). These considerations call for sustained reflexive awareness of the interpretive and provisional character of clinician first-person inquiry.

## 5. Conclusions

Attending to the therapist’s lived experience may add a clinically useful dimension to psychodynamic understanding, particularly when similar symptom presentations generate different relational and affective demands. Such experience may provide a source of working clinical hypotheses requiring reflection, consideration alongside the unfolding therapeutic material, and, where possible, supervision. A psychodynamic–phenomenological approach may support a more reflective use of therapist subjectivity while preserving the relational and interpretive character of clinical understanding.

## Figures and Tables

**Table 1:** Comparative synthesis of the two clinical cases from a psychodynamic–phenomenological perspective.

Dimension	Case One (Narcissistic Personality Traits)	Case Two (Obsessive–Compulsive Personality Traits)
Presenting concerns	Longstanding anxiety, wish for greater self-understanding and a more secure sense of self, episodes of overwhelming stress	Progressive anxiety, anticipatory worry, panic-like episodes, emotional exhaustion
Dominant affective organization	Shame, narcissistic vulnerability, unstable self-esteem	Guilt, harsh superego, excessive responsibility
Core relational conflict	Longing for recognition while fearing dependency and humiliation	Longing for support while fearing criticism, weakness, and moral failure
Predominant defenses	Withdrawal, devaluation, idealization, factualization, control	Intellectualization, overcontrol, suppression of anger, moralization, turning aggression against the self
Resistance	Emotional contact repeatedly interrupted by distancing and reduction in affect	Reflection interrupted by affective flooding, silence, and self-criticism
Transference	Therapist experienced as a valued object whose increasing importance risked making dependency more visible	Therapist experienced as a potentially judging or disappointed authority
Countertransference	Pressure to remain useful and preserve recognition without intensifying dependency	Pressure to preserve reflective functioning without intensifying self-attack
Therapist’s lived experience	Uncertainty, discouragement, fatigue, bodily caution, recurring sense that emotional contact was slipping away	Vigilance, protective responsibility, careful pacing, concern about overwhelming the patient, urge to regulate affect
Therapeutic atmosphere	Delicate, intermittent, emotionally elusive; closeness repeatedly faded	Emotionally intense, constricted, carefully regulated; affect threatened to exceed containment
Capacity to symbolize and reflect on affect	Affect generally remained thinkable, but intimacy became difficult to sustain	Reflective capacity rapidly diminished as affect intensified, leading to crying, bodily tension, or freezing
Technical emphasis	Maintaining relational continuity while minimizing shame and defensive retreat	Providing containment, regulating pace, and supporting symbolization before interpretation
Overall psychodynamic understanding	Anxiety embedded within narcissistic vulnerability and regulation of self-esteem	Anxiety embedded within obsessive–compulsive overcontrol, severe superego functioning, and chronic guilt

## Data Availability

The underlying clinical material is not publicly available owing to patient confidentiality and privacy considerations.

## Note

1 Glossary of key psychodynamic concepts. *Transference* refers to the reactivation of established relational patterns within the therapeutic relationship. *Countertransference* refers to the therapist’s emotional, bodily, and relational responses arising within the therapeutic encounter. *Object relations* refers to internalized patterns of experiencing the self in relation to significant others. *Shame* concerns experiences of exposure, inadequacy, humiliation, or threats to self-worth, whereas *guilt* concerns perceived wrongdoing, responsibility, or failure to meet internalized moral demands. *Defensive organization* refers to characteristic psychological processes for managing anxiety, conflict, and difficult affects. *Developmental conflict* refers to enduring tensions among needs, wishes, prohibitions, and relational demands shaped through earlier developmental experience. *Affective symbolization* refers to the capacity to transform emotional and bodily experience into representations that can be recognized, reflected upon, and communicated. *Superego functioning* refers to internalized standards, prohibitions, and self-evaluative demands, particularly as expressed through guilt and self-criticism.
